# GLS-409, an Antagonist of Both P2Y_1_ and P2Y_12_, Potently Inhibits Canine Coronary Artery Thrombosis and Reversibly Inhibits Human Platelet Activation

**DOI:** 10.1038/s41598-018-32797-1

**Published:** 2018-09-28

**Authors:** Elena Smolensky Koganov, Alan D. Michelson, Ivan B. Yanachkov, Milka I. Yanachkova, George E. Wright, Karin Przyklenk, Andrew L. Frelinger

**Affiliations:** 1 Center for Platelet Research Studies, Dana-Farber/Boston Children’s Cancer and Blood Disorders Center, Harvard Medical School, Boston, MA USA; 2 GLSynthesis Inc., Worcester, MA USA; 3 Cardiovascular Research Institute and Departments of Physiology and Emergency Medicine, Wayne State University School of Medicine, Detroit, MI USA

## Abstract

Dual antiplatelet therapy with aspirin and an adenosine diphosphate (ADP) P2Y_12_ receptor antagonist reduces ischemic events in patients with acute coronary syndrome. Previous evidence from our group, obtained in a preclinical model of recurrent platelet-mediated thrombosis, demonstrated that GLS-409, a diadenosine tetraphosphate derivative that inhibits both P2Y_1_ and P2Y_12_ ADP receptors, may be a novel and promising antiplatelet drug candidate. However, the salutary antiplatelet effects of GLS-409 were accompanied by a trend toward an unfavorable increase in bleeding. The goals of this study were to: 1) provide proof-of-concept that the efficacy of GLS-409 may be maintained at lower dose(s), not accompanied by an increased propensity to bleeding; and 2) establish the extent and kinetics of the reversibility of human platelet inhibition by the agent. Lower doses of GLS-409 were identified that inhibited *in vivo* recurrent coronary thrombosis with no increase in bleeding time. Human platelet inhibition by GLS-409 was reversible, with rapid recovery of platelet reactivity to ADP, as measured by platelet surface activated GPIIb-IIIa and platelet surface P-selectin. These data support the concept that GLS-409 warrants further, larger-scale investigation as a novel, potential therapy in acute coronary syndromes.

## Introduction

The current mainstay of pharmacological therapy for preventing ischemic events in patients with acute coronary syndrome (ACS), including those undergoing percutaneous coronary intervention, is dual antiplatelet therapy with aspirin and an inhibitor of the platelet adenosine-5′-diphosphate (ADP) receptor, P2Y_12_^1–4^. Newer P2Y_12_ inhibitors (prasugrel, ticagrelor and cangrelor) produce greater and more consistent platelet inhibition and reduce ischemic events to a greater degree than clopidogrel. However, these agents are associated with significantly increased bleeding risk^2,5–8^. Moreover, despite the introduction of these new drugs and the use of dual antiplatelet therapy, many patients continue to have recurrent atherothrombotic events^3,4,9^.

Platelets express two purinergic receptors that respond to ADP: P2Y_1_ and P2Y_12_. There is a complex interplay between P2Y_1_ and P2Y_12_^10^, and co-activation of both receptors is required for full platelet aggregation^11^. However, all of the currently FDA-approved ADP receptor inhibitors (ticlopidine, clopidogrel, prasugrel, ticagrelor and cangrelor) target only the P2Y_12_ receptor. We recently reported synthesis of new diadenosine tetraphosphate (Ap_4_A) base- and polyphosphate chain-substituted derivatives with simultaneous inhibitory activity at both P2Y_1_ and P2Y_12_ receptors, resulting in synergistic inhibition of platelet aggregation^12,13^. Further investigation of one of these Ap_4_A derivatives GLS-409 (see Supplementary Fig. S1) demonstrated rapid inhibition of *in vitro* agonist-stimulated platelet aggregation following intravenous infusion in rats, rapid improvement in coronary patency in a canine model of *in vivo* platelet-mediated thrombosis, and a short plasma half-life^12,13^. These characteristics suggest that rapid, reversible simultaneous inhibition of P2Y_1_ and P2Y_12_ with GLS-409 may be a useful treatment modality during the initial phase of ACS when atherothrombotic risk and bleeding risk are both high. Thus, GLS-409 is envisioned as an early treatment for patients in need of antithrombotic therapy, with the benefit of rapid onset of inhibition and short plasma half-life^12–14^, allowing protective platelet inhibition to be initiated quickly, yet also allowing platelet inhibition to be quickly discontinued under emergent conditions.

GLS-409 at a dose of 0.054 mg/kg IV bolus followed by a continuous intravenous infusion of 0.0018 mg/kg/min, attenuated recurrent platelet-mediated thrombosis and significantly improved coronary patency in the classic canine model that mimics human unstable angina^12^. However, this salutary effect of GLS-409 on vessel patency was accompanied by a modest but potentially unfavorable, 30% increase in median template bleeding time^12^. Accordingly, the first objective of the current study was to provide proof-of-concept that the efficacy of GLS-409 may be maintained at lower dose(s), not accompanied by an increased propensity to bleeding. In addition, to gain insight into the recovery of platelet function after discontinuation of GLS-409 therapy, our second objective was to examine the extent and kinetics of the reversibility of platelet inhibition by GLS-409 added *in vitro* to the blood of healthy human subjects.

## Results

### Effect of GLS-409 on coronary patency in a canine model of recurrent coronary thrombosis

Using a ‘delayed’ treatment study design, animals were assigned to receive either GLS-409 (n = 13) or matched volumes of vehicle (saline; n = 3) initiated at 1 hour after the onset of recurrent coronary thrombosis. Three doses of GSL-409 were evaluated: 1) 0.054 mg/kg bolus + 0.00018 mg/kg/min infusion maintained for 2 hours (same bolus + 1/10 of the infused dose administered in the initial GLS-409 study, n = 3)^12^; 2) 0.0054 mg/kg bolus + 0.00018 mg/kg/min infusion for 2 hours (1/10 of the bolus + 1/10 of the infused dose administered in the initial study, n = 5); or 3) 0.00054 mg/kg bolus + 0.000018 mg/kg/min infusion for 2 hours (1/100 of the bolus + 1/100 of the infused dose administered in the initial study, n = 5).

**Figure 1:**
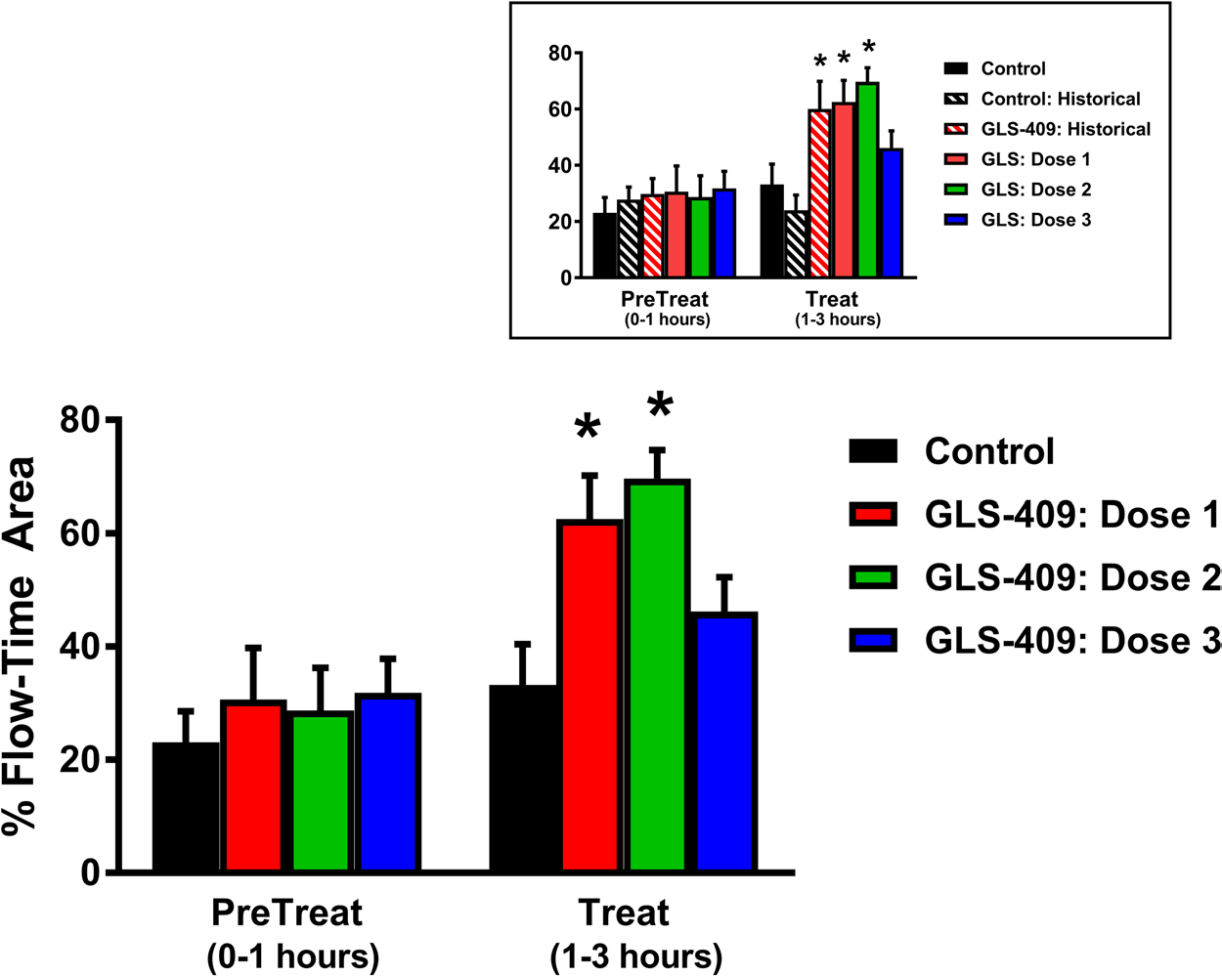
Effect of GLS-409 and vehicle control (saline) on coronary patency as measured by % flow-time area in a canine model of recurrent arterial thrombosis. % Flow-time area, quantified before and after treatment, in cohorts treated with saline (Control n = 3) and GLS-409 doses 1, 2 and 3. Dose 1: 0.054 mg/kg bolus + 0.00018 mg/kg/min infusion maintained for 2 hours (same bolus + 1/10 of the infused dose administered in the initial GLS-409 study, n = 3)^12^. Dose 2: 0.0054 mg/kg bolus + 0.00018 mg/kg/min infusion for 2 hours (1/10 of the bolus + 1/10 of the infused dose administered in the initial study, n = 5). Dose 3: 0.00054 mg/kg bolus + 0.000018 mg/kg/min infusion for 2 hours (1/100 of the bolus + 1/100 of the infused dose administered in the initial study: n = 5). Insert: for purposes of comparison, data from the current study are plotted together with results obtained previously with high-dose GLS-409 (0.054 mg/kg IV bolus followed by a continuous intravenous infusion of 0.0018 mg/kg/min) and matched (historical) controls^12^. Data are mean ± SEM, *p < 0.05 versus pretreatment and p < 0.05 versus controls.

**Figure 2:**
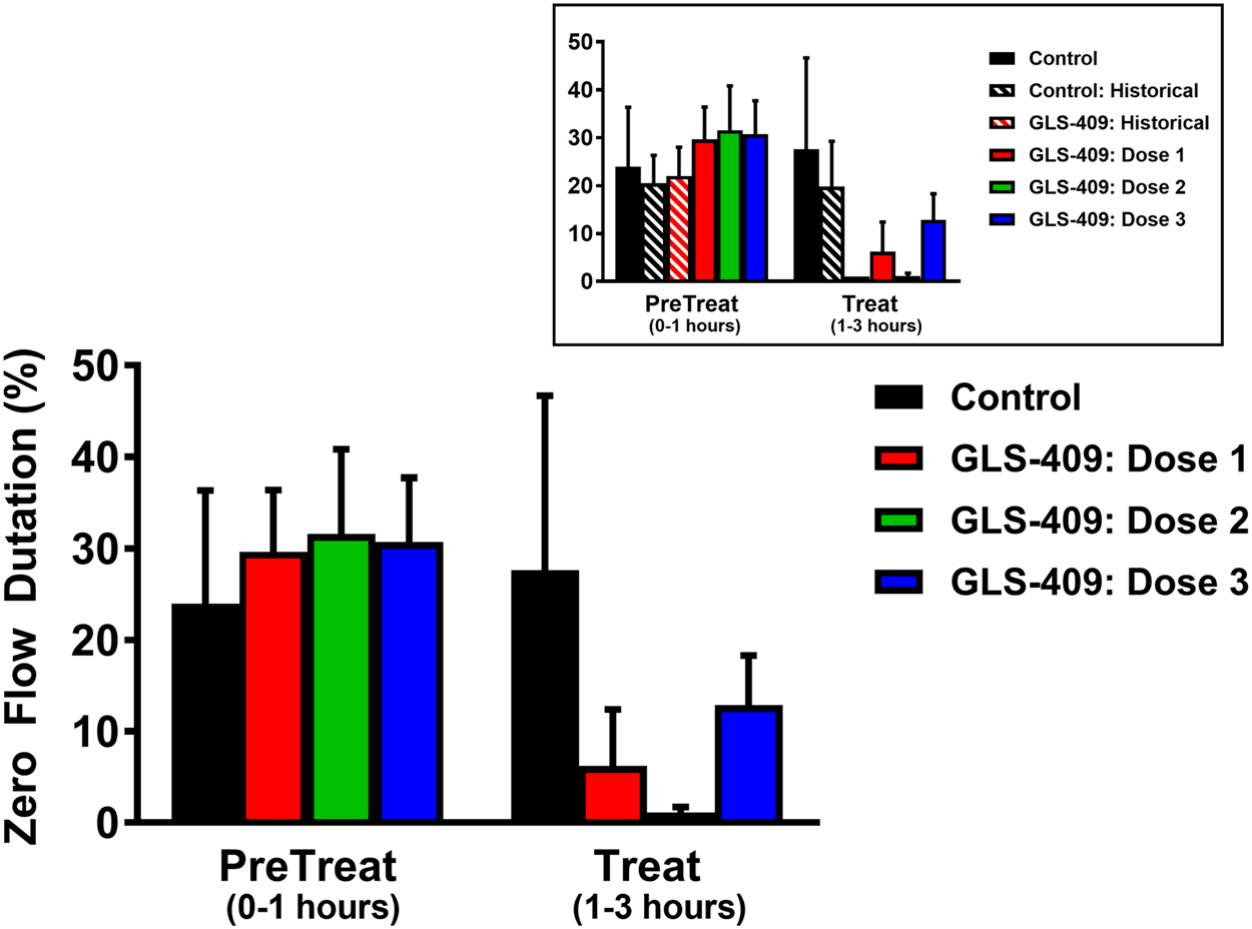
Effect of GLS-409 and vehicle control (saline) on coronary patency as measured by zero flow duration in a canine model of recurrent arterial thrombosis. % Zero flow duration (mean ± SEM), quantified before and after treatment, in cohorts treated with saline (Control) and GLS-409 doses 1, 2 and 3 (see Fig. 1 legend for details). p = 0.14 (ns) for group-time interaction. Insert: for purposes of comparison, data from the current study are plotted together with results obtained previously with high-dose GLS-409 (0.054 mg/kg IV bolus followed by a continuous intravenous infusion of 0.0018 mg/kg/min) and matched (historical) controls^12^.

**Figure 3:**
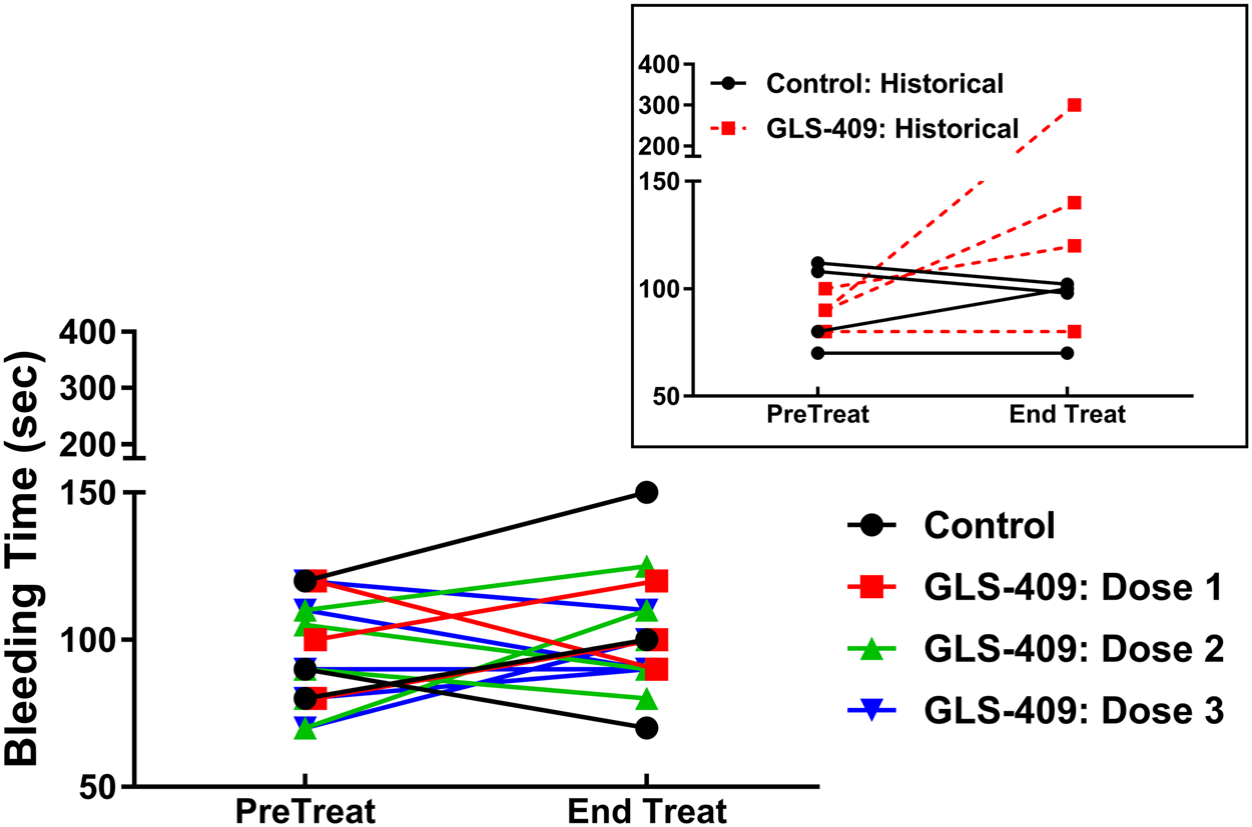
Effect of GLS-409 and vehicle control (saline) on the template bleeding time. Bleeding time (seconds), measured immediately before treatment and at the end of the 2-hour treatment period, for cohorts treated with saline (Control) and GLS-409 Doses 1, 2 and 3 (see Fig. 1 legend for details). Insert: for purposes of comparison, results obtained previously with high-dose GLS-409 (0.054 mg/kg IV bolus followed by a continuous intravenous infusion of 0.0018 mg/kg/min) and matched (historical) controls are shown^12^.

Coronary patency before randomization and treatment was comparable in all 4 cohorts: mean flow-time area and zero flow duration ranged from 23–32% (Fig. 1) and 24–32% (Fig. 2), respectively. In addition, the template bleeding times (median 90–100 sec, ranges 70–125 sec) assessed before treatment with drug or vehicle were similar for all groups (Fig. 3). In the saline control group, there was no change in coronary patency during the 2-hour treatment period when compared with the pretreatment phase (Figs 1 and 2). Administration of GLS-409 at doses 1 and 2 was associated with significant increases in % flow-time area (to 63 ± 8% and 70 ± 5%, respectively, p < 0.05 *versus* control, Fig. 1; for a representative tracing of coronary flow results and matched pre-treat and treat flow-time area results see Supplementary Figs S2 and S3) that were comparable in magnitude to the results obtained with the original, high dose of the agent (Fig. 1 insert)^12^. This was accompanied by a trend (p = 0.14 for group-time interaction) toward an attenuation in zero flow duration (Fig. 2). However, and in contrast to the outcomes obtained previously with high-dose GLS-409^12^, the better maintenance of coronary patency achieved with GLS-409 doses 1 and 2 was not confounded by increases in the template bleeding time (Fig. 3). The lowest dose of GLS-409 (dose 3) had no significant effect on % flow-time area and did not affect the template bleeding time (Figs 1 and 3). Finally, and as anticipated from the data obtained with the high-dose of GLS-409^12^, doses 1, 2 and 3 administered in the current protocol had no effect on the heart rate or arterial pressure (data not shown).

### Recovery of human platelet reactivity following 30 minutes exposure to GLS-409

**Figure 4:**
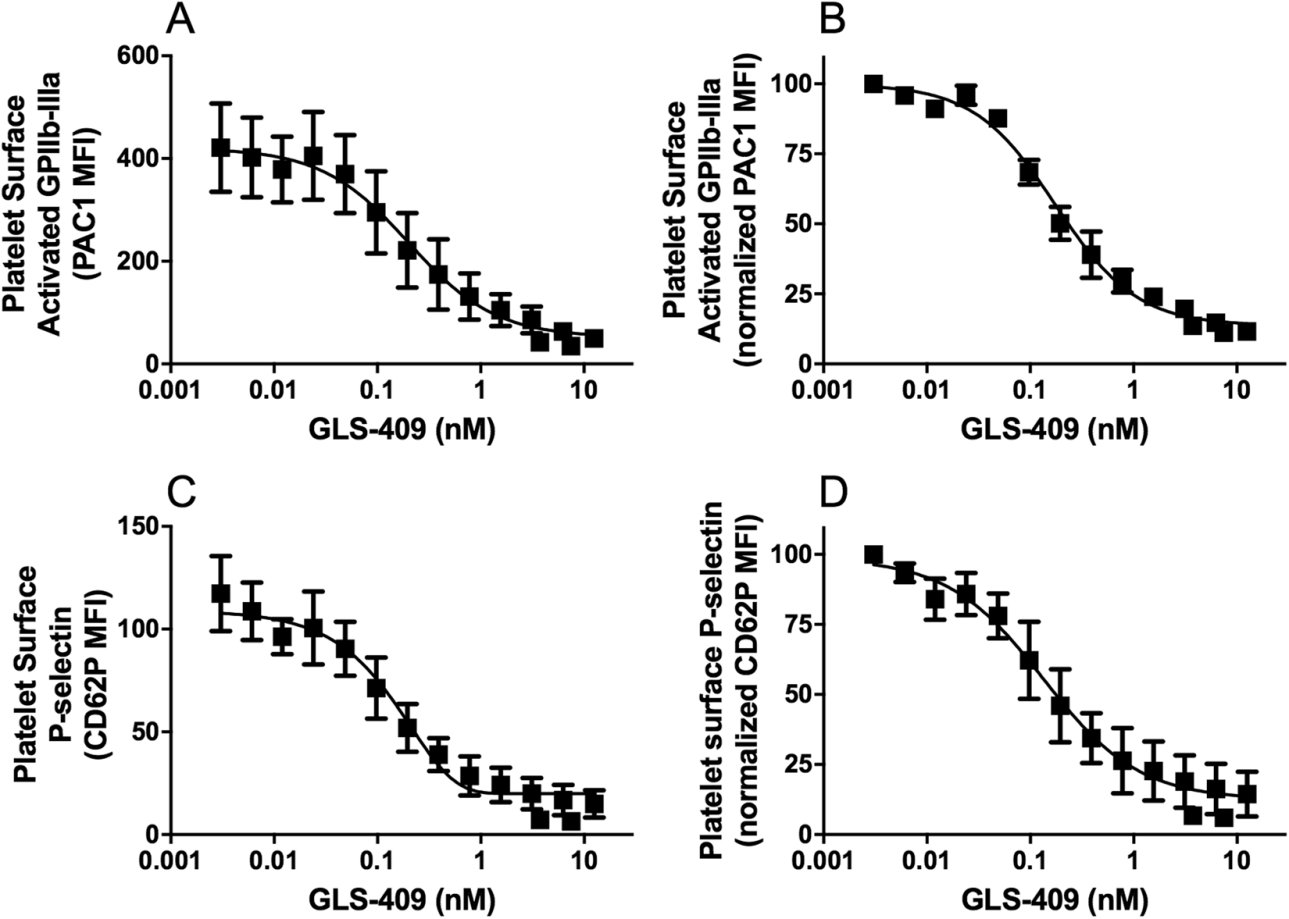
GLS-409 concentration-dependent inhibition of human platelet reactivity to ADP as measured by platelet surface activated GPIIb-IIIa and platelet surface P-selectin expression. Sodium citrate 3.2% anticoagulated whole blood from healthy volunteers was treated *in vitro* with different concentrations of GLS-409 (0.003–12.5 nM) or vehicle. (**A**) Platelet surface activated GPIIb-IIIa, detected with the activation-dependent monoclonal antibody PAC1 (MFI, mean fluorescence intensity) and (**B**) Normalized MFI of PAC1. IC_50_ = 0.17 nm (95% CI, 0.13–0.22), R^2^ = 0.97. (**C**) MFI and (**D**) normalized MFI for platelet surface P-selectin. IC_50_ = 0.13 nM (95% CI, 0.065–0.25), R^2^ = 0.84. Data were analyzed using fitting of non-linear regression. Results are mean ± SEM, n = 3.

**Figure 5:**
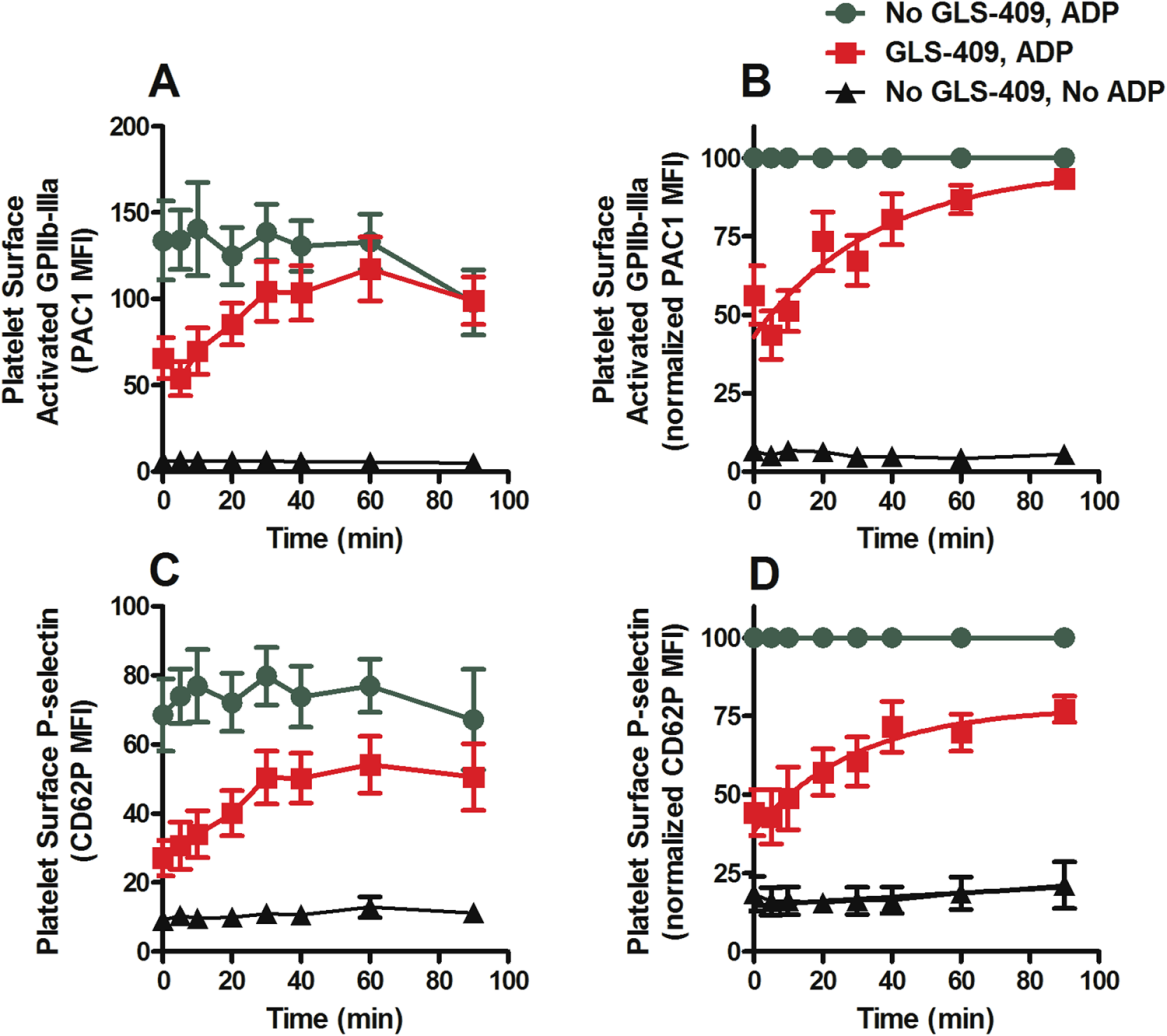
Reversibility of human platelet inhibition by GLS-409: recovery of platelet reactivity to ADP. Sodium citrate 3.2% anticoagulated whole blood from healthy volunteers was treated with vehicle (saline) or the IC_80_ dose (1.56 nm) of GLS-409 for 30 minutes at room temperature. Treated blood was diluted 300-fold in drug-free filtered platelet-poor plasma and then incubated at room temperature for different lengths of time (0–90 min), followed by ADP 5 µM stimulation and measurement of platelet surface activated GPIIb-IIIa and platelet surface P-selectin. (**A**) MFI and (**B**) normalized MFI of PAC1, as a percentage of maximal response at each point, for platelet surface activated GPIIb-IIIa. (**C**) MFI and (**D**) normalized MFI, as a percentage of maximal response at each point, for platelet surface P-selectin. In panels B and D, data are expressed as the percentage of the control (vehicle treatment) at the same time point, and data were analyzed using non-linear fit of dissociation: one-phase exponential decay in GraphPad Prism. Data are mean ± SEM, n = 9.

Sodium citrate 3.2% anticoagulated whole human blood exposed *in vitro* to GLS-409 for 30 min at room temperature showed a concentration-dependent inhibition of ADP-stimulated platelet surface activated GPIIb-IIIa and P-selectin (Fig. 4) with 50% inhibition obtained at ~ 0.17 nM GLS-409. Subsequent experiments to characterize the reversibility of platelet inhibition by GLS-409 were performed using the IC_80_ concentration of GLS-409, 1.56 nM, in order to obtain strong, consistent platelet inhibition. Figure 5 shows the time dependence of the recovery of platelet reactivity to ADP, after 30 min incubation of whole blood with the IC_80_ dose (1.56 nM) of GLS-409, and then 300-fold dilution of the treated whole blood in drug-free platelet-poor plasma. Figure 5A,C show that there is a slight but consistent decrease over time in the platelet response to ADP, due to sample aging. To exclude this factor, and reduce the variability between the time points, the results were normalized as a percentage of maximal response at each point (Fig. 5B,D), of platelet surface activated GPIIb-IIIa (reported as MFI of PAC1) and platelet surface P-selectin. The time required after exposure of blood to GLS-409 for 50% recovery of ADP-stimulated PAC1 reactivity was ~26 min (Fig. 5A,B). At 60 min after the 300-fold dilution of samples, activated GPIIb-IIIa on ADP-stimulated platelets was not statistically different from that of ADP-stimulated results of vehicle-treated samples, indicating full recovery of platelet reactivity to ADP (Fig. 5A,B). The recovery following 300-fold dilution of GLS-409 of ADP-stimulated platelet surface activated GPIIb-IIIa to the same levels as vehicle-treated controls suggests any inhibition of platelet aggregation would also be fully reversed. In contrast, even at 90 min after dilution in drug-free plasma, ADP-stimulated platelet surface P-selectin expression showed only ~75% recovery (Fig. 5C,D) and was still significantly different from the vehicle-treated control.

## Discussion

In the present study we report two findings. First, we provide proof-of-concept that the favorable, *in vivo* inhibition of recurrent platelet-mediated thrombosis with the novel compound GLS-409 can be achieved at doses that are not accompanied by an increase in the template bleeding time. Second, we demonstrate that human platelet inhibition by GLS-409 was fully reversible (T_1/2_ ~26 min) as measured by ADP-stimulated changes in platelet surface activated GPIIb-IIIa and partially reversible as measured by ADP-stimulated changes in platelet surface P-selectin. These properties of GLS-409, combined with its previously demonstrated simultaneous inhibition of both P2Y_1_ and P2Y_12_ receptors^13^ and short plasma half-life^12^, suggest that this novel agent may have the potential to be of particular therapeutic benefit during the initial phase of ACS when atherothrombotic risk and bleeding risk are both high.

Previous *in vitro* binding studies showed that ticagrelor binds reversibly to the P2Y_12_ receptor^15,16^. More rapid recovery of function of existing platelets would be expected following discontinuation of ticagrelor, compared with clopidogrel or prasugrel which bind irreversibly to P2Y_12_, and whose recovery of platelet function therefore depends on regeneration of platelets. However, after discontinuation of ticagrelor, platelet inhibition persists for several days including times when plasma concentrations of ticagrelor are undetectable^15,17,18^. We recently demonstrated that following 24 hrs of exposure of platelets to ticagrelor, platelet inhibition as measured by ADP-stimulated platelet surface activated GPIIb-IIIa was only partially reversible^16^, providing a possible explanation for the delay in platelet function recovery after exposure to ticagrelor. Because ticagrelor, a triazolopyrimidine analogue of ATP^19^, and GLS-409, a modified Ap_4_A^13^, are so structurally dissimilar, it cannot be predicted whether prolonged exposure of platelets to GLS-409 would, like ticagrelor, lead to incomplete reversibility. However, in this study we demonstrate full reversibility of ADP-stimulated surface activated GPIIb-IIIa on platelets, after exposure to GLS-409 for 30 min. The time required after *in vitro* exposure of platelets to GLS-409 for 50% recovery of platelet reactivity as measured by ADP-stimulated binding of PAC1 (~26 min) is longer for GLS-409 than that observed in our previous study with cangrelor (1.8 min), ticagrelor (4.4 min), and the ticagrelor active metabolite (6.3 min)^16^. The relatively slow reversibility of GLS-409 inhibition (compared to ticagrelor and cangrelor) may offer another explanation, in addition to its dual specificity for P2Y_1_ and P2Y_12_ receptors, for the presently-described high potency of GLS-409 observed in preclinical models. After exposure of platelets to GLS-409 for 30 min, the complete recovery of platelet function as measured by ADP-stimulated activated GPIIb-IIIa and the partial (~75%) recovery of platelet function as measured by ADP-stimulated platelet surface P-selectin, was similar to our recently-reported results for cangrelor^16^, which also shows incomplete reversibility of ADP-stimulated P-selectin, but different from ticagrelor, which showed complete recovery of both markers^16^. The mechanisms underlying incomplete reversibility of ADP-stimulated platelet surface P-selectin with GLS-409 and cangrelor remain unclear, but given that cangrelor acts on P2Y_12_ but not P2Y_1_, this effect is unlikely to be due to GLS-409’s inhibition of P2Y_1_.

Recent observations show that hemostatic thrombi formed in response to a penetrating injury are composed of a dense core of fibrin-associated platelets overlaid by a shell of more loosely packed, less activated platelets^20–23^. Experiments with P2Y_12_ antagonists disrupted mainly the cap while leaving the dense core of platelets largely intact^20–23^. Whether combined inhibition of P2Y_1_ and P2Y_12_ by GLS-409 allows greater selectivity for the cap relative to the core is unknown. However, the synergy seen by inhibition of both P2Y_1_ and P2Y_12_ with respect to platelet aggregation is likely to be recapitulated in limiting platelet accumulation in arterial thrombi.

Currently, combined antiplatelet therapy with aspirin and an inhibitor of the platelet ADP receptor P2Y_12_ reduces the risk of ischemic events in patients with ACS. However, those events still occur, thereby raising the question of whether inhibition of other pathways of platelet activation would be clinically beneficial. GLS-409 is envisioned for use during the initial phase of ACS, by administration of the drug early in the clinical encounter with the patient when acute myocardial infarction is suspected but prior to a final diagnosis, with the goal of reducing cardiovascular damage. Moreover, intravenous GLS-409 could be used during angiography and percutaneous coronary intervention, including stent placement, followed by oral antiplatelet therapy with aspirin and a P2Y_12_ inhibitor. If coronary artery bypass graft surgery will be required, GLS-409 can be discontinued and, due to its short plasma half-life^12^, rapid clearance of GLS-409 would allow rapid recovery of platelet function, thereby shortening the delay to surgery and reducing the bleeding risk associated with platelet inhibition. If angiography reveals that the patient does not have ACS, discontinuation of GLS-409 will allow rapid recovery of platelet function.

In conclusion, GLS-409 targets both P2Y_1_ and P2Y_12_ receptors and may therefore provide a more complete inhibition of ADP-induced platelet aggregation. The presently-demonstrated proof-of-concept evidence of *in vivo* inhibition of platelet-mediated arterial thrombosis by GLS-409 without an associated increase in the bleeding time, together with our data documenting reversibility of human platelet inhibition by GLS-409, suggest that this novel agent may be of benefit during the initial phase of ACS when the atherothrombotic risk and bleeding risk are both high. Accordingly, further evaluation of the potential clinical utility of GLS-409 is warranted.

## Materials and Methods

### Drugs and materials

#### Canine model of recurrent coronary thrombosis

GLS-409 was provided by GLSynthesis Inc., Worcester, MA. Ketamine, midazolam and isoflurane were purchased from Zoetis, Parsippany, NJ, Hospira, Cudahy, WI and VetOne, Boise, ID, respectively.

#### Recovery of platelet reactivity following 30 minutes exposure to GLS-409

GLS-409 was provided by GLSynthesis Inc., Worcester, MA. Stocks of GLS-409 compound, diluted in HEPES-saline buffer were maintained at −80 °C. The antibody cocktail included phycoerythrin (PE)-conjugated anti-P-selectin monoclonal antibody, (CD62P, clone AK-4, BD Pharmingen, San Jose, CA), fluorescein isothiocyanate (FITC)-conjugated monoclonal antibody PAC1 (BD Biosciences, San Jose, CA), glycoprotein (GP) IIb-IIIa, and PE-Cy5–conjugated anti-CD42b (GPIb) monoclonal antibody (BD-Pharmingen, San Jose, CA). The isotype control included PE-Cy5–conjugated anti-CD42b (GPIb) monoclonal antibody (BD-Pharmingen, San Jose, CA), PE-conjugated MIgG1 isotype (BD Pharmingen, San Jose, CA), FITC-PAC1 (BD Biosciences, San Jose, CA), and the GPIIb-IIIa antagonist Integrilin (eptifibatide, Millennium Pharmaceuticals, Cambridge, MA), to block specific FITC-PAC1 binding. Platelets stimulation was achieved by addition of ADP 5 µM (Bio/Data Corporation, Horsham, PA). The reaction was stopped by addition of 300 µL of 1% formaldehyde in HEPES-saline buffer.

### Canine model of recurrent coronary thrombosis

#### Ethics Statement

Experiments conducted in the canine model were approved by the Institutional Care and Use Committee of Wayne State University (Protocol A 01-02-14), and performed in accordance with the *Guide for the Care and Use of Laboratory Animals*^24,25^.

#### Surgical preparation

Sixteen female adult Class A purpose-bred mongrel dogs (weight: 14–24 kg) were anesthetized with ketamine + midazolam (33 mg/kg IM + 0.1–0.5 mg/kg IM) and inhaled isoflurane (1–4%) and instrumented as described in our initial study investigating the effect of GLS-409 on recurrent thrombosis^12^. Briefly, catheters were positioned in the left jugular vein and in the left carotid artery (for administration of fluids and hemodynamic monitoring, respectively), and the heart was exposed via a left lateral thoracotomy. Two adjacent segments of the left anterior descending coronary artery (LAD) were then isolated and the distal segment was instrumented with a Doppler flow probe (Transonic Systems Inc., Ithaca, NY) for continuous measurement of mean coronary blood flow, and the proximal segment served as the site of later thrombosis. Importantly, there was only one procedural change: continued use of sodium pentobarbital, the anesthetic utilized in the previous protocol^12^, had become impractical because of escalating costs and limited availability. After stabilization, recurrent coronary thrombosis was initiated using standard methods described previously^12,26–28^. Specifically, the proximal LAD segment was compressed with forceps to induce endothelial denudation and medial injury. A micromanometer constrictor was then positioned at the site of injury and tightened such that mean coronary blood flow was reduced to 30–35% of its baseline value, triggering the rapid onset of cyclic variations in coronary blood flow (cyclic flow variations, CFVs) caused by platelet activation-aggregation and the resultant spontaneous accumulation- dislodgment of platelet-rich thrombi at the site of injury + stenosis (see Supplementary Fig. S2). At 1 hour after the onset of recurrent thrombosis, dogs were assigned to receive either GLS-409 (n = 13) or matched volumes of vehicle (saline; n = 3). Three doses of GLS-409 were assessed: 1) 0.054 mg/kg bolus + 0.00018 mg/kg/min infusion maintained for 2 hours (same bolus + 1/10 of the infused dose administered in the initial GLS-409 study, n = 3)^12^; 2) 0.0054 mg/kg bolus + 0.00018 mg/kg/min infusion for 2 hours (1/10 of the bolus + 1/10 of the infused dose administered in the initial study, n = 5); or 3) 0.00054 mg/kg bolus + 0.000018 mg/kg/min infusion for 2 hours (1/100 of the bolus + 1/100 of the infused dose administered in the initial study, n = 5). Coronary blood flow was monitored throughout the 2 hours post-treatment. At the conclusion of the protocol, animals were euthanized under deep anesthesia with Fatal-Plus (0.22 ml/kg IV).

#### Endpoints and analysis

Endpoints (including coronary patency following injury + stenosis, the template bleeding time, and hemodynamics (heart rate and arterial pressure)) were measured as described previously^12^. The primary endpoint, coronary patency, was assessed by quantifying two variables: the duration of total thrombotic occlusion (‘zero flow duration’ *i*.*e*., coronary blood flow [CBF] = 0 mL/min); and ‘flow-time area’, defined as the area of the flow-time tracing divided by the baseline coronary flow^26–28^. Zero flow duration and flow-time area measured during each phase of the protocol (before *versus* after randomization and treatment) were normalized and expressed as a % of the respective observation time (60 minutes *versus* 120 minutes)^28^.

### Recovery of platelet reactivity following 30 minutes exposure to GLS-409

#### Ethics statement and subjects

This study was approved by the Boston Children’s Hospital Institutional Review Board and conducted in accordance with the Declaration of Helsinki. Signed informed consent was obtained from each subject, prior to participation in this study. Freshly drawn venous blood from healthy volunteers, who had not taken any antiplatelet medications within the previous 14 days, was collected into 3.2% sodium citrate anticoagulated tubes.

#### Determination of GLS-409 IC_80_

Sodium citrate 3.2% anticoagulated whole blood from healthy volunteers (n = 3) was treated *in vitro* with different concentrations of GLS-409 (0.003–12.5 nM) or vehicle, incubated for 30 min and then assessed for ADP-stimulated platelet surface activated GPIIb-IIIa and P-selectin by flow cytometry as previously described^16,29,30^ and detailed below.

#### Recovery of platelet reactivity following 30 minutes exposure to GLS-409

Sodium citrate 3.2% anticoagulated whole blood from healthy volunteers (n = 9) was treated with vehicle, or the IC_80_ dose of GLS-409 for 30 minutes at room temperature. A portion of the citrate anticoagulated whole blood without GLS-409 treatment was centrifuged (5000 g, 5 min) and the supernatant filtered with 0.2 μm filters (VWR, Radnor, PA) to generate drug-free platelet-poor plasma (PPP). After 30 min treatment of citrate anticoagulated whole blood with vehicle or GLS-409, samples were diluted 300-fold in drug-free filtered PPP (thereby reducing the concentration of GLS-409 to 0.0052 nM) and incubated at room temperature for different times (aliquots taken at 0, 5, 10, 20, 30, 40, 60 and 90 min), followed by ADP 5 µM stimulation (15 min) and measurement of platelet surface activated GPIIb-IIIa and platelet surface P-selectin. The antibody cocktail included PE-conjugated CD62P anti-P-selectin monoclonal antibody, FITC-conjugated monoclonal antibody PAC1, which only binds to the activated conformation of GPIIb-IIIa, and PE-Cy5–conjugated anti-CD42b (GPIb) monoclonal antibody. The isotype control included PE-Cy5–conjugated anti-CD42b (GPIb) monoclonal antibody, phycoerythrin (PE)-conjugated MIgG1 isotype FITC-PAC1, and the GPIIb-IIIa antagonist eptifibatide (to block specific FITC-PAC1 binding). The reaction was stopped by addition of 300 µL of 1% formaldehyde in HEPES-saline buffer. Flow cytometric analyses were performed as previously described^29^ in a FACSCalibur flow cytometer (Becton Dickinson) which was calibrated daily. Established voltages for forward scatter (FSC) and side scatter (SSC) were used to present platelets in the middle of the dot plot and staining with an antibody against CD42b-PE-Cy5 was used to further identify platelets. Activated GPIIb-IIIa and P-selectin were measured relative to the isotype control as percentage positive cells and as mean fluorescence intensity (MFI) before and after 300-fold dilution in drug-free platelet-poor plasma.

#### Statistical analysis

Data were analyzed using GraphPad Prism, versions 5 and 7 (San Diego, CA). Endpoints in the canine model were compared by 2-factor ANOVA (for group and time) with replication, and results are presented as mean ± SEM. Results were considered significant if *P* < 0.05. The data for the recovery of platelet reactivity were fitted using dissociation-one phase exponential decay with the formula Y = (Y_0_ − NS) * exp (−K*X) + NS (where Y_0_ is the Y value when X is zero, NS is the Y value at infinite times and K is the rate constant). IC_50_s are expressed as mean followed by 95% confidence interval (95% CI).

## Electronic supplementary material

Supplementary Information
