# Publisher Correction: GLS-409, an Antagonist of Both P2Y_1_ and P2Y_12_, Potently Inhibits Canine Coronary Artery Thrombosis and Reversibly Inhibits Human Platelet Activation

**DOI:** 10.1038/s41598-018-35956-6

**Published:** 2018-11-29

**Authors:** Elena Smolensky Koganov, Alan D. Michelson, Ivan B. Yanachkov, Milka I. Yanachkova, George E. Wright, Karin Przyklenk, Andrew L. Frelinger

**Affiliations:** 1 Center for Platelet Research Studies, Dana-Farber/Boston Children’s Cancer and Blood Disorders Center, Harvard Medical School, Boston, MA USA; 2 GLSynthesis Inc., Worcester, MA USA; 3 Cardiovascular Research Institute and Departments of Physiology and Emergency Medicine, Wayne State University School of Medicine, Detroit, MI USA

Correction to: *Scientific Reports* 10.1038/s41598-018-32797-1, published online 28 September 2018

In the original version of this Article, the author Elena Smolensky Koganov was incorrectly indexed. This error has now been corrected.

