# Supplementary material for: GLS-409, an Antagonist of Both P2Y1 and P2Y12, Potently Inhibits Canine Coronary Artery Thrombosis and Reversibly Inhibits Human Platelet Activation
Source: Sci Rep. 2018 Sep 28;8:14529. doi: 10.1038/s41598-018-32797-1 (PMC6162268; doi:10.1038/s41598-018-32797-1)
Supplement: Supplementary file 1 — Supplementary Information [file 41598_2018_32797_MOESM1_ESM.docx]

**GLS-409, an Antagonist of Both P2Y1 and P2Y12, Potently Inhibits Canine Coronary Artery Thrombosis and Reversibly Inhibits Human Platelet Activation**

Elena Smolensky Koganov^1^, Alan D. Michelson^1^, Ivan B. Yanachkov^2^, Milka I. Yanachkova^2^, George E. Wright^2^, Karin Przyklenk^3^ and Andrew L. Frelinger III^1^

^1^Center for Platelet Research Studies, Dana-Farber/Boston Children's Cancer and Blood Disorders Center, Harvard Medical School, Boston, MA; ^2^GLSynthesis Inc., Worcester, MA; ^3^Cardiovascular Research Institute and Departments of Physiology and Emergency Medicine, Wayne State University School of Medicine, Detroit, MI

**Supplemental Material**

**Supplemental Figure S1. Chemical structure of GLS-409.**

**
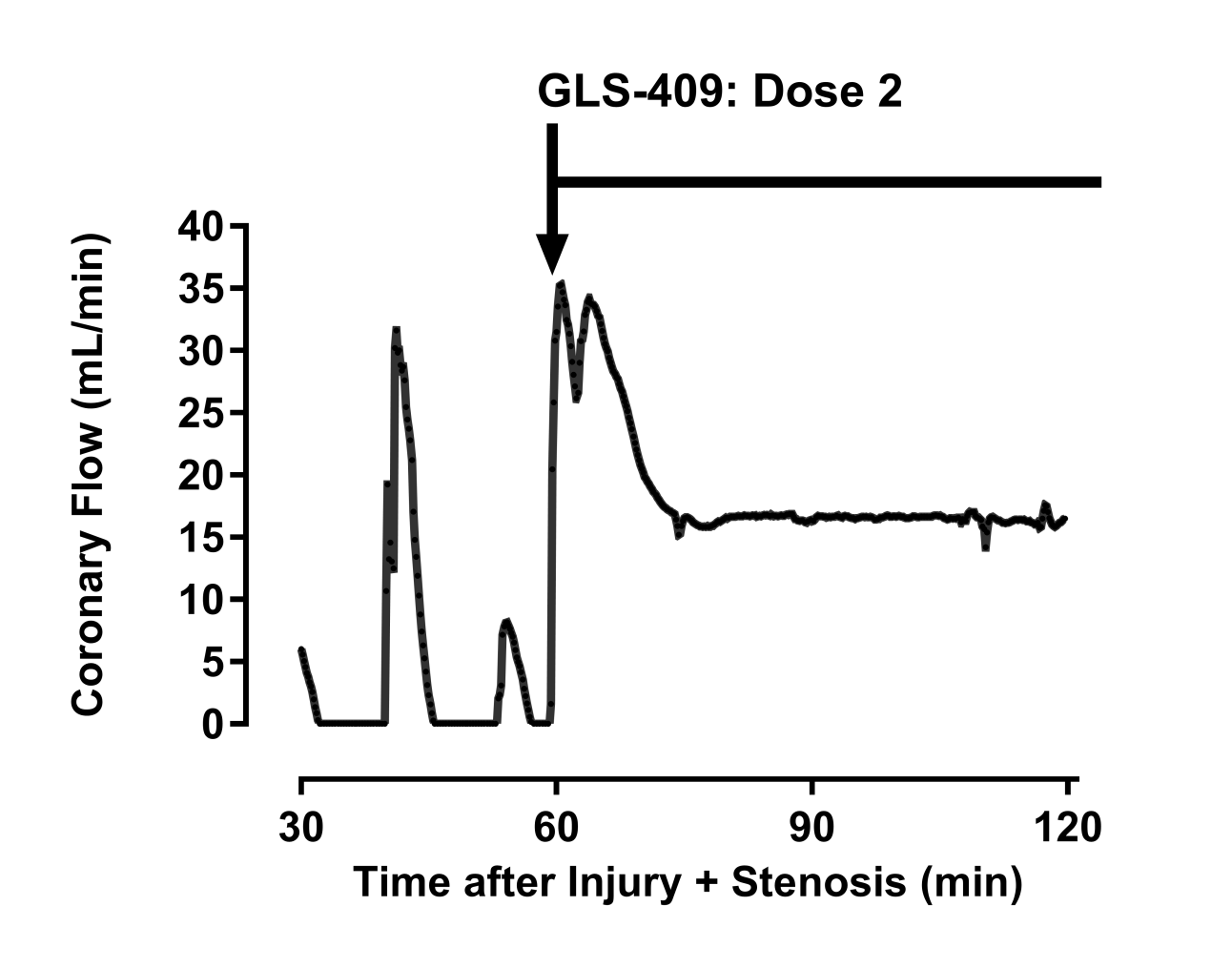
**

**Supplemental Figure S2.** Representative tracing of coronary blood flow in a canine model of recurrent arterial thrombosis before and after administration of GLS-409. Coronary blood flow, measured following coronary artery injury + stenosis. Representative results for one animal treated with Dose 2 of GLS-409 (0.0054 mg/kg bolus + 0.00018 mg/kg/min infusion for 2 hours). Coronary injury + stenosis initiated the spontaneous formation-dislodgment of platelet-rich thrombi, manifest as cyclic variations in coronary flow (CFVs). Administration of GLS-409 (bolus + infusion denoted by the arrow + line) was associated with better maintenance of coronary patency.

**Supplemental Figure S3. Effect of GLS-409 and vehicle control (saline) on coronary patency as measured by % flow-time area in a canine model of recurrent arterial thrombosis: Individual matched results.** Results presented in Figure 1 of the main manuscript are re-plotted here to show results from individual animals. Dose 1: 0.054 mg/kg bolus + 0.00018 mg/kg/min infusion maintained for 2 hours (same bolus + 1/10 of the infused dose administered in the initial GLS-409 study)^12^. Dose 2: 0.0054 mg/kg bolus + 0.00018 mg/kg/min infusion for 2 hours (1/10 of the bolus + 1/10 of the infused dose administered in the initial study). Dose 3: 0.00054 mg/kg bolus + 0.000018 mg/kg/min infusion for 2 hours (1/100 of the bolus + 1/100 of the infused dose administered in the initial study).
